# Targeting STAT3 and NF-κB Signaling Pathways in Cancer Prevention and Treatment: The Role of Chalcones

**DOI:** 10.3390/cancers16061092

**Published:** 2024-03-08

**Authors:** Violetta Krajka-Kuźniak, Marta Belka, Katarzyna Papierska

**Affiliations:** Department of Pharmaceutical Biochemistry, Poznan University of Medical Sciences, Rokietnicka 3, 60-806 Poznan, Poland; mbelka@ump.edu.pl (M.B.); kpapierska@ump.edu.pl (K.P.)

**Keywords:** chalcones, STAT signaling pathway, NF-κB signaling pathway, cancer prevention, chalcones nanoformulations, cancer treatment

## Abstract

**Simple Summary:**

Chalcones are natural compounds with proven biological properties, including anticancer activities. However, their low bioavailability encourages the design of new synthetic derivatives whose modified structure would increase the possibilities of use in the therapy or prevention of cancer. The prepared literature review presents natural and synthetic derivatives of chalcones that can modulate the NF-κB and STAT3 signaling pathways, the excessive activation of which plays an essential role during the carcinogenesis of various types of cells. Recent in vitro and in vivo research suggest tremendous therapeutic potential for natural and synthetic chalcone derivatives as compounds in multiple combinations and nanoformulations demonstrating better anticancer effects.

**Abstract:**

Chalcones are a type of natural flavonoid compound that have been found to possess promising anticancer properties. Studies have shown that chalcones can inhibit the growth and proliferation of cancer cells, induce apoptosis, and suppress tumor angiogenesis. In addition to their potential therapeutic applications, chalcones have also been studied for their chemopreventive effects, which involve reducing the risk of cancer development in healthy individuals. Overall, the anticancer properties of chalcones make them a promising area of research for developing new cancer treatments and preventative strategies. This review aims to provide a thorough overview of the central studies reported in the literature concerning cancer prevention and the treatment of chalcones. Although chalcones target many different mechanisms, the STAT and NF-κB signaling pathways are the ones this review will focus on, highlighting the existing crosstalk between these two pathways and considering the potential therapeutic opportunities for chalcone combinations.

## 1. Introduction

Cancer is a complex disease that arises from the accumulation of genetic mutations and alterations in cellular pathways. Emerging evidence suggests that chronic inflammation plays a crucial role in the development and progression of various types of cancer [1]. Chalcones, a class of natural compounds found in many plants, have been shown to possess potent anti-inflammatory and anticancer properties. Moreover, several studies have demonstrated that synthetic chalcones exhibit potent anticancer effects through multiple mechanisms [2]. Chalcones exhibit a versatile and multifaceted mode of action by influencing various signaling pathways implicated in the development and progression of cancer. Their ability to modulate key cellular processes makes them promising candidates for anticancer therapies. Chalcones have been shown to impact pathways involved in cell proliferation, apoptosis, angiogenesis, and metastasis, collectively contributing to their anticancer properties. However, the specific emphasis on the signal transducer and activator of transcription (STAT) and nuclear factor-kappa B (NF-κB) pathways is justified by the central roles these pathways play in tumorigenesis. Literature reviews confirm that both natural and synthetic chalcones inhibit the activation of key signaling pathways, such as STAT and NF-κB involved in inflammation and cancer. By inhibiting these pathways, chalcones can suppress the growth and survival of cancer cells, induce apoptosis, and sensitize cancer cells to chemotherapy and radiation therapy. Several studies have reported the anticancer effects of chalcones on various types of cancer, including breast, lung, colon, and prostate cancer [3]. These findings highlight the potential of chalcones as a novel class of anticancer agents targeting key signaling pathways involved in cancer development and progression. 

In this overview, we present the results from studies based on databases (PubMed, Scopus, Web of Science, and Google Scholar), which were searched using the terms: “chalcones”, “STAT”, and “NF-κB”. This review aims to underscore the therapeutic possibilities of natural and synthetic chalcones, indicating their potential points of action—the STAT3 and NF-κB pathways.

## 2. Structures and Mechanism of Action of Chalcones

**Figure 1:**
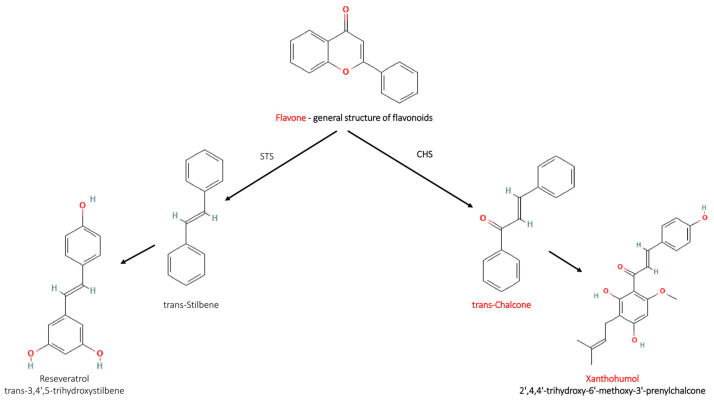
Biosynthesis pathway of flavonoids and stilbene derivatives, and chalcone derivatives. STS (stilbene synthase) and CHS (chalcone synthase) are enzymes that facilitate the synthesis of trans-stilbene and trans-chalcone from flavonoids [Chemical Structure Records from PubChem].

Chalcones are a group of plant-derived polyphenolic compounds belonging to the flavonoid family. Flavonoids occur in plants and act as dyes, antioxidants, natural insecticides, and fungicides, protecting against attacks by insects and fungi. The chemical structure of flavonoids is based on a 2-phenylchroman (flavan) or 3-phenylchroman (isoflavan) skeleton, with most types of flavonoids (except catechins and anthocyanidins) containing a flavone or isoflavone skeleton, with a ketone group in position 4 (Figure 1). Flavonoids differ in the number and type of substituents; the differences between compounds in individual classes usually result from the different structures of only one extreme ring [4]. Chalcones originate from the phenylpropanoid pathway, with p-coumaroyl-CoA, derived from phenylalanine, serving as the initial precursor. The second precursor, malonyl-CoA, is obtained through the malonic acid pathway.

Further reactions, as presented in Figure 1, depend on the activity of chalcone synthase (CHS) or stilbene synthase (STS), respectively, which results in the formation of chalcones (C6-C3-C6) or stilbenes (C6-C2-C6). STS and CHS are homodimeric, related plant-specific polyketide synthases. Both enzymes perform a sequential condensation of three acetate units to a starter residue to form an intermediate that is folded to the ring systems specific to the different products of resveratrol and chalcone’s natural derivatives, such as piceatannol, polydatin, viniferin, and pinocembrin chalcone [5].

**Figure 2:**
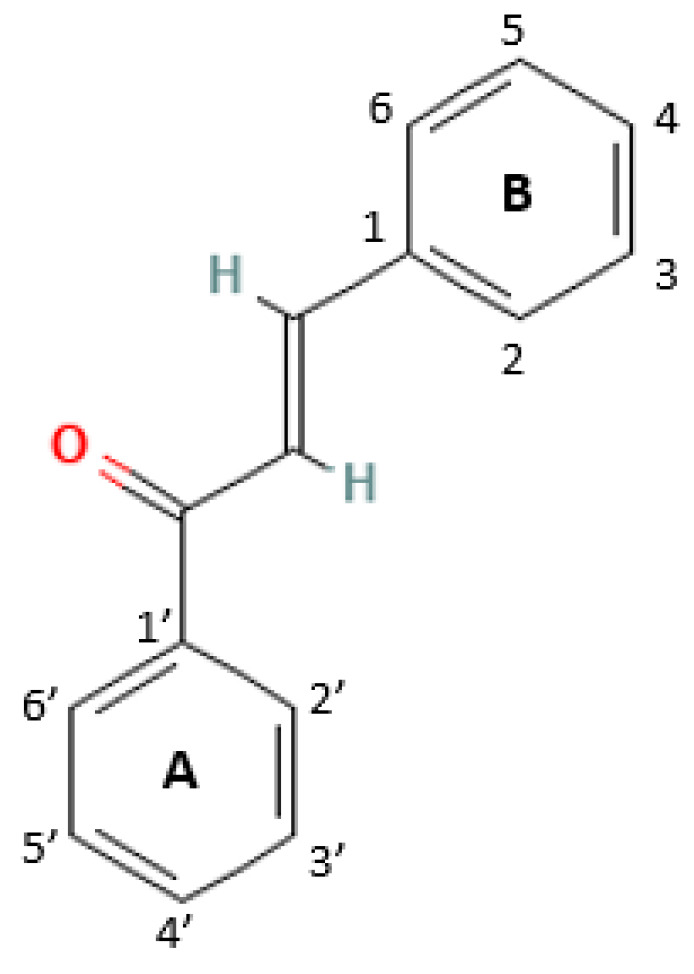
The chemical structure of chalcone. The essential property of the chemical structure of chalcones is an open chain with three carbon molecules bonded to the A and B rings [Chemical Structure Records from PubChem].

Chalcones consist of two aromatic rings, typically a benzene ring (phenyl ring) and another aromatic ring, connected by a three-carbon α,β-unsaturated carbonyl system (Figure 2). 

This unique structure is often called the “enone bridge” and is the defining feature of chalcones. The general formula of chalcones can be represented as R_1_-C6H4-C(O)-CH=CH-C6H4-R_2_, where R_1_ and R_2_ represent various substituents or functional groups. The α,β-unsaturated carbonyl system in the molecule’s center imparts reactivity and contributes to the biological activities exhibited by chalcones.

Natural chalcones exhibit many biological activities, including anticancer, anti-angiogenic, anti-inflammatory, antioxidant, immunomodulatory, anti-bacterial, anti-malarial, and analgesic [3,6,7]. Chalcones can induce apoptosis, cell cycle arrest, or inhibit tumor promotion and metastasis in cancer cells, e.g., breast, hematopoietic system, or reproductive organs [8,9]. They are also effective as chemopreventive agents related to various properties such as antioxidant, anti-inflammatory, anti-bacterial, anticancer, cytotoxic, and immunosuppressive. Moreover, compounds from this family have been shown to interfere with every step of carcinogenesis, including initiation, promotion, and progression [10,11]. These biological activities are mainly attributed to the presence of an α,β-unsaturated carbonyl system in the chalcones, perceived as a potential acceptor of the Michael reaction (leading to the forming of a new carbon–carbon bond) [12]. This group can easily form covalent bonds with nucleophiles, such as thiol cysteine residues in peptides or cell proteins, to obtain a Michael adduct that may play an essential role in the molecule’s biological activity [13].

A good safety profile and the possibility of oral administration are the main factors contributing to the growing interest in the study of the therapeutic potential of chalcones. It has been shown that the oral administration of 3-nitro-2-hydroxy-4,6-dimethoxychalcone to a group of animals infected with *Leishmania infantum* or *Leishmania amazonensis* is not only safe but also brings the expected effects [14]. Moreover, obtaining the desired results after the oral administration of chalcones is also confirmed by studies involving patients, an example of which is a double-blind, placebo-controlled study involving 92 people. It was then shown that the oral intake of cyclohexenyl chalcone derivatives (panduratin A) for 12 weeks significantly increases skin hydration and shine and reduces wrinkles without adverse symptoms [15].

Therefore, new chalcone derivatives are still being sought, whose structure, improved by chemical synthesis, could show a broader or more specific spectrum of activity toward human cells [16]. 

Natural chalcones have attracted particular attention from scientists due to their wide range of biological activity and potential therapeutic use. Unfortunately, they do not accumulate in plants in large quantities because they are used as intermediates for the biosynthesis of flavonoids, which generates a problem with obtaining them in the necessary amounts [16,17]. 

The limitations of using natural chalcones are their low solubility in water, poor absorption, and short residence time in the intestine. For these reasons, research is being conducted on modifying the molecular structure of these compounds, which would allow for obtaining derivatives with better physicochemical and biological properties [16,18].

The relationship between the structure of chalcones and their anticancer activity has been widely studied. Chalcones are a class of natural or synthetic compounds that consist of two aromatic rings connected by a three-carbon α,β-unsaturated carbonyl system. The structural variations in chalcones, such as substitutions on the aromatic rings or modifications in the linker region, have significantly impacted their anticancer properties. These modifications can alter the physicochemical properties of chalcones, including lipophilicity, molecular size, and electronic distribution, which ultimately affect their interactions with cellular targets and biological activities [19].

Chalcones can occur in the form of two isomers: cis and trans. The trans isomer is the dominant configuration because it is most often thermodynamically more stable. In order to change the biological activity of molecules, the chemical structure of 1,3-diphenyl-2-propen-1-one can be modified by replacing hydrogens with functional groups, for example: phenyl, carboxylic, aryl, hydroxyl, or halogen [20]. Synthetic chalcones are most often obtained by Claisen–Schmidt condensation. The synthesis methods also include the Heck, Sonogashira, or Suzuki–Miyaura coupling reaction and the reaction involving an acid catalyst [21,22].

One of the key factors influencing the anticancer activity of chalcones is their ability to interfere with various cellular signaling pathways that play crucial roles in tumor growth and progression. Chalcones, natural compounds found in plants, have demonstrated the capacity to modulate signaling pathways involved in cancer development and metastasis. Some prominent signaling pathways affected by chalcones include the STAT and NF-κB pathways.

Chalcones have been reported to exhibit a wide range of anticancer effects, including the inhibition of cell proliferation, the induction of apoptosis (programmed cell death), the suppression of angiogenesis (formation of new blood vessels), and the modulation of immune responses [20].

The presence of specific functional groups on the chalcone structure, such as hydroxyl, methoxy, or nitro groups, has been correlated with enhanced anticancer activity. These groups can interact with specific molecular targets within cancer cells, such as enzymes or receptors, and disrupt their normal function. For example, chalcones with hydroxyl groups have been found to inhibit key enzymes involved in cell proliferation, while those with methoxy groups have shown potent anti-angiogenic effects [23].

Moreover, the substitution pattern on the aromatic rings of chalcones can influence their interactions with cellular transporters and drug efflux pumps, affecting their bioavailability and resistance mechanisms. By modifying the structure of chalcones, researchers aim to optimize their pharmacokinetic properties and overcome drug resistance, ultimately improving their anticancer efficacy [24].

Several studies have pointed out that methoxy substitutions on aryl rings and an increase in the compound’s lipophilicity are essential for improving its anticancer activity. 

In conclusion, the structure–activity relationship of chalcones plays a crucial role in their anticancer potential. Understanding the impact of structural modifications on the biological activity of chalcones can guide the design and development of novel compounds with improved efficacy and selectivity against cancer cells.

## 3. STAT and Chalcones

### 3.1. STAT Signaling Pathways as a Target

STAT (signal transducer and activator of transcription) proteins are a family of transcription factors that play a crucial role in cellular signaling and gene expression. They transmit signals from the cell surface to the nucleus, where they regulate the expression of specific target genes. STAT proteins are activated in response to various extracellular signals, such as growth factors, cytokines, and hormones [25]. The canonical STAT activation pathway is typically initiated by ligand binding to specific cell surface receptors, activating receptor-associated kinases and the subsequent phosphorylation of specific tyrosine residues, e.g., Tyr705, on the receptor itself. Once phosphorylated, the receptor serves as a docking site for cytoplasmic STAT proteins. The STAT proteins are latent transcription factors that exist in an inactive state in the cytoplasm in the absence of stimulation [26]. The phosphorylation of STAT proteins on specific tyrosine residues induces their conformational changes, promoting their dimerization through reciprocal interactions between their SH2 (Src homology 2) domains. The dimerized STAT proteins then translocate to the nucleus, where they bind to specific DNA sequences known as STAT response elements (SREs) located in the promoter regions of target genes. The binding of STAT dimers to SREs leads to the recruitment of coactivators and chromatin-modifying complexes, resulting in the transcriptional activation of target genes [27]. The canonical STAT activation pathway is highly regulated, involving multiple feedback mechanisms to ensure the precise control of gene expression. Negative regulators such as protein tyrosine phosphatases and suppressors of cytokine signaling (SOCS) proteins can dephosphorylate STATs or inhibit their activation, while protein inhibitors of activated STATs (PIAS) can block their DNA-binding and transcriptional activity [28].

While the canonical pathway involves the binding of cytokines to their respective receptors and the subsequent activation of Janus kinases (JAKs) leading to STAT phosphorylation, the non-canonical activation of STAT can occur through alternative mechanisms. One example of non-canonical STAT activation is mediated by receptor tyrosine kinases (RTKs). RTKs, such as the epidermal growth factor receptor (EGFR) or platelet-derived growth factor receptor (PDGFR), can directly phosphorylate and activate STAT proteins in response to ligand binding. These ligands can activate downstream signaling pathways involving phosphoinositide 3-kinase (PI3K) and Src kinases, which phosphorylate and activate STATs independently of JAKs [29]. G protein-coupled receptors (GPCRs), which are involved in diverse cellular processes, can also trigger non-canonical STAT activation. GPCR activation can induce intracellular signaling cascades leading to the activation of nonreceptor tyrosine kinases, such as JAK family members or Src family kinases, which phosphorylate STATs. Non-canonical STAT activation through GPCRs has been observed in different situations, such as inflammation and cancer [30]. The other mechanism of non-canonical STAT activation is DNA damage response. DNA double-strand breaks can trigger the activation of ataxia-telangiectasia mutated (ATM) and DNA-dependent protein kinase (DNA-PK), which phosphorylate and activate STAT proteins independently of cytokine receptors. The non-canonical activation of STAT1 and STAT3 in response to DNA damage has been observed and is implicated in DNA repair and cellular stress responses. Other studies demonstrated that toll-like receptors (TLRs) activation can lead to the non-canonical activation of STATs through the involvement of nonreceptor tyrosine kinases, such as spleen tyrosine kinase (Syk) and Lyn (Lck/Yes-related novel protein tyrosine kinase), or downstream signaling components like MyD88 (Myeloid differentiation primary response 88) and TRIF (TIR-domain-containing adapter-inducing interferon-β). The non-canonical activation of STAT1 and STAT3 by TLRs has been reported in immune cells, contributing to the regulation of immune responses [31,32]. 

### 3.2. Chalcones as STAT Inhibitors

Interest in chalcones as compounds potentially inhibiting STAT proteins is constantly growing. The abnormal activation of STAT signaling has already been linked to a variety of diseases, including cancer, inflammation, and autoimmune disorders. The inhibition of STAT proteins has emerged as a promising therapeutic strategy for targeting these diseases [33]. 

**Figure 3:**
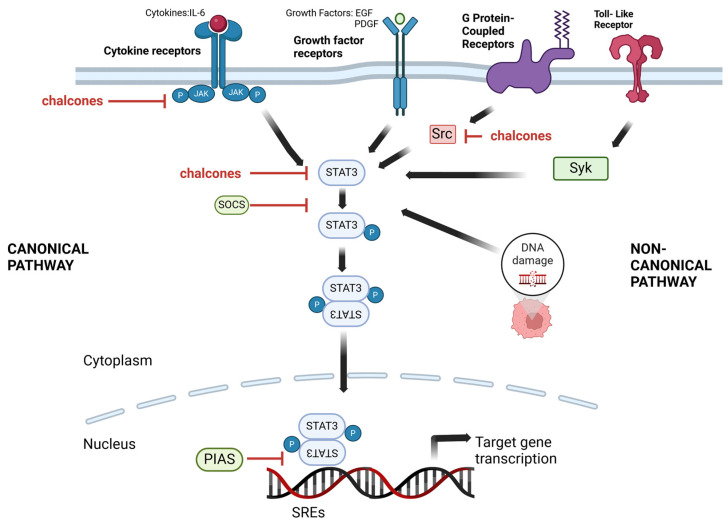
The proposed mechanism of chalcones on the inhibition of STAT3. The left part of the figure represents the canonical pathway of STAT3 activations; the right part is the non-canonical pathway of STAT3 activation. Chalcones can inhibit the STAT3 pathway by interfering with the phosphorylation of STAT3 proteins and inhibiting kinases’ activity, such as JAK and Src. The figure was created using information from the references given in Section 3 (STAT and chalcones). EGF, epidermal growth factor; IL-6, interleukin-6; JAK, Janus kinases; P, phosphorylation; PDGFR, platelet-derived growth factor; PIAS, protein inhibitors of activated STATs; SOCS, protein tyrosine phosphatases and suppressors of cytokine signaling; Src, Src kinases; SREs, STAT response elements; STAT3, signal transducer and activator of transcription; Syk, spleen tyrosine kinase; TLRs, toll-like receptors. Created with BioRender.com.

Based on extensive databases (PubMed, Scopus, Web of Science, and Google Scholar), several studies have explored the potential of chalcones as STAT inhibitors (Table 1) by targeting different steps of the STAT3 signaling pathway, as presented in Figure 3.

One of the mechanisms through which chalcones inhibit STAT3 activation directly interferes with the phosphorylation of STAT3 proteins. Phosphorylation is critical for STAT activation, leading to its dimerization, nuclear translocation, and subsequent transcriptional activity. Most chalcones listed in Table 1 impact STAT3 phosphorylation in vitro and in vivo models. Many studies have confirmed that the common phosphorylation site of STAT3 is tyrosine at position 705. This effect was observed for natural chalcones such as xanthohumol [34], cardamonin [35], licochalcone C [36], licochalcone D [37], licochalcone H [38], phloretin [39], butein [40], and isoliquiritigenin [41]. 

Similar effects were exhibited with synthetic chalcones like (E)-3-(4-bromo-3,5-dimethoxyphenyl)-1-(3-hydroxyphenyl)prop-2-en-1-one [42], (E)-1–(1-hydroxy-4,5,8-trimethoxynaphthalen-2-yl)-3-(quinolin-6-yl) prop-2-en-1-one [43], 4,3′,4′,5′-tetramethoxychalcone [44], (E)-3-(7-(3,4-dimethoxyphenyl)-2-phenylpyrazolo[1,5-a]pyrimidin-5-yl)-1-(3,4,5-trimethoxyphenyl)prop-2-en-1-one [45], (E)-1-(2,4-dimethoxyphenyl)-3-(4-hydroxy-3,5-dimethoxyphenyl)prop-2-en-1-one [46], ZE-2-(4-(4-chlorophenyl)-6-(4-nitrophenyl)pyrimidin-2-ylthio)-N-(4-(3-(3,4-dimethoxyphenyl)acryloyl)phenyl)acetamide [47], and 3-(4-methylthiophene)-1-(3-bromo-4,5-dimethoxyphenyl)prop-2-en-1-on [48].

Attention should also be paid to the organ-specific dependence on STAT3 phosphorylation. This phenomenon has been predominantly documented in breast cancers [49,50], oral cancers [36,37], and liver cancers [39,40,51,52].

However, the team of Wu et al. described the mechanism of phosphorylation STAT3 using docking analysis for cardamonin [53]. They documented that STAT3 dimerization relies on SH2 domains connected to a loop (from Ala-702 to Phe-716) originating from its monomers. Specifically, Leu-706, Thr-708, and Phe-710 mechanically engage with a cavity on the SH2 domain of the other monomer in conjunction with phosphorylated Tyr-705. Small molecules that bind selectively to this cavity are anticipated to hinder STAT3 dimerization by competing with the amino acid residues. Docking results suggested that cardamonin could establish several hydrogen bonds with Ser-613, Arg-609, and Lys-591, implying a direct interaction between cardamonin and STAT3 [53].

Fascinating studies were also conducted by the team of Dong et al. involving demonstrating the effectiveness of STAT3 inhibition through a benzochalcone derivative, e.g., (E)-1–(1-hydroxy-4,5,8-trimethoxynaphthalen-2-yl)-3-(quinolin-6-yl) prop-2-en-1-one, using the molecular docking method. They showed that a reciprocal pY705-SH2 domain interaction mediates the homodimerization of STAT3. In the STAT3 SH2 domain, three adjacent binding subpockets were explored for drug targeting, including (1) the phosphorylated Tyr705 (p-Tyr705)-binding pocket (also named pY subpocket, residues 591, 609–620); (2) the Leu706 subsite (also named pYþ1 subpocket, residues 626–639); and (3) a side pocket (also named pY-X subpocket, residues 592–605). Among them, the pY site is the most important for inhibitor binding to disrupt STAT3 phosphorylation and dimerization. The molecular modeling experiment revealed that the hydrogen bond was a predominant factor for this benzochalcone derivative tightly binding to STAT3 [43].

As mentioned earlier, kinases are essential in the STAT phosphorylation process. The literature data indicate that chalcones have been shown to inhibit the activation of STAT3 proteins by inhibiting the activity of kinases, such as JAK [54]. Studies on licochalcones, especially licochalcone D, confirmed this, showing the involvement of JAK2 kinase and its phosphorylation at Tyr 1007/1008 [37]. A similar effect was noted for cardamonin in the in vitro and in vivo models [35]. Additionally, in the case of licochalcone B, it was indicated that the inhibition of JAK2 kinase occurs through the binding of chalcone to the catalytic domain of this kinase. Moreover, the inhibition of JAK2 kinase was dose-dependent with this chalcone [55].

Also, in case experiments including incubation isoliquiritigenin with human renal carcinoma Caki cells, the inhibition of binding STAT3 to DNA results from the phosphorylation of STAT3 at both Y705 and S727 residues was observed. Consequently, isoliquiritigenin attenuated the constitutive phosphorylation of JAK2 and the expression of STAT3 target gene products such as cyclin D1 and cyclin D2 [56].

Many studies have indicated that chalcones modulate the expression of negative regulators of the STAT pathway, such as SOCS proteins (suppressors of cytokine signaling). These proteins act as negative feedback regulators by binding to activated STATs and promoting their ubiquitination and degradation. In turn, chalcones can increase the expression of SOCS proteins, thereby attenuating STAT signaling [57]. The literature data indicate that only cardamonin has been analyzed among the described chalcones in the context of its impact on SOCS proteins. Research by Zhang et al. showed that cardamonin did not affect the protein levels of SOCS-1 or SOCS-3 in prostate cancer cell lines (DU145) [58].

However, research by Ning et al. [59] showed that isoliquiritigenin induced the protein inhibition of activated signal transducer and activators of transcription 3 (PIAS3) in breast cancer cell lines (Hs-578T, MDA-MB-231). Additionally, the abrogation of PIAS3 via the transfection of specific siRNA abolished the inhibitory effect of isoliquiritigenin on the activity of the STAT3 signaling pathway and miR-21 expression. This study documented for the first time that the induction of PIAS3-mediated STAT3 signaling inhibition was responsible for the repression of miR-21 by isoliquiritigenin. However, the issue of STAT and microRNA relationships requires more extensive research.

In the case of the non-canonical STAT activation pathway, JAK-independent kinases such as ERK and Src kinases are responsible for the phosphorylation of STAT. Studies by Rajendran et al. [40] have shown that butein can inhibit STAT activation by affecting both JAK2 and Src kinases. However, in the case of cardamomin, only the effect on JAK2 kinase was noted, while there was no effect on Src kinase in the prostate cancer cell line (DU145) [58]. Among the synthetic chalcones discussed, 4,3′,4′,5′-tetramethoxychalcone decreased kinase Src in ovarian cancer cell lines (A2780 and SKOV3) [44] and N-(4-(3-(4-methoxyphenyl)acryloyl)phenyl)-2-((5-(3,4,5-trimethoxy-phenyl)-1,3,4-oxadiazol-2-yl)thio)acetamide in leukemia cell lines (K-562) [60].

The translocation of STAT3 from the cytosol to the nucleus and, consequently, its binding to DNA was demonstrated for butein [61], cardamonin [58], and isoliquiritigenin [56], using EMSA assay.

Notably, the activation of the STAT pathway can lead to both the activation and inhibition of various genes, which affects a variety of cellular processes such as proliferation (e.g., myelocytomatosis, c-Myc), differentiation (e.g., cyclin D1), cellular survival (e.g., B-cell lymphoma-extra large, Bcl-xl; Bcl-2-associated X protein, Bax; survivin; Myeloid cell leukemia 1, Mcl-1), angiogenesis (e.g., vascular endothelial growth factor, VEGF), and immune responses (interleukin-6, IL-6). Jiang’s research showed that xanthohumol downregulated the expression of target genes such as Bcl-xL, cyclin D1, and survivin in pancreatic cancer BxPC-3 and PANC-1 cells [34]. Xanthohumol dose-dependently decreased Bcl-xl levels and increased Bax levels in breast cancer cell lines (MCF-7) [50], pancreatic cancer cell lines (PANC-1) [34], and choliangiocarcinoma cell lines (M139 and M214) [51].

Likewise, cardamonin hindered crucial proteins regulated by STAT in the glioblastoma stem cell line (CD133+). Wu et al. illustrated that, at a dosage of 40 µM, cardamonin suppressed the phosphorylation of IL-6-induced STAT3. Furthermore, cardamonin reduced the levels of anti-apoptotic proteins (Bcl-xL, Mcl-1, survivin), cell cycle-regulatory protein (cyclin D1), and VEGF in a manner dependent on the dosage [53]. Similar effects were observed with cardamomin, albeit in a different cell line, specifically, the prostate cancer cell line (DU145). In this case, cardamonin demonstrated a time-dependent decrease in the expression of Bcl-xl, Bcl-2, survivin, VEGF, cyclin D1, and cyclin E proteins [58].

Also, butein has been extensively tested and has been shown to inhibit the expression of Bcl-xL, Bcl-2, cyclin D1, and Mcl-1 in multiple myeloma cell lines (U266) [61], and the expression of cyclin D1, Bcl-2, Bcl-xL, survivin, and VEGF in a liver cancer cell line (HepG2) [40].

In summary, the potential of chalcones as STAT inhibitors has been investigated in various in vivo studies and cell-based assays, as shown in Table 1. These studies have demonstrated the ability of chalcones to inhibit STAT activation, suppress downstream gene expression, and exhibit antiproliferative effects in cancer cells. However, further research is needed to determine their efficacy and safety in preclinical studies and to optimize their chemical structure for improved potency and selectivity.

**Table 1:** Summary of natural and synthetic chalcones acting on STAT3 pathways, the model used, and results obtained.

Name of Chalcones	In Vitro/In Vivo Models	Effect on STAT3	References
Butein *Semecarpus anacardium*	liver cancer cell line (HepG2)	↓ phosphorylation of STAT3 ↓ nuclear translocation of STAT3	[40]
	multiple myeloma cell line (U266)	↓ phosphorylation of STAT3 ↓ STAT3 DNA-binding activity	[61]
	lung cancer cell line (A549) breast cancer cell line (MDA-MB-231)	↓ phosphorylation of STAT3	[49]
Cardamonin *Alpinia rafflesiana*	colon cancer cell lines (HT-29 and SW-460)	↓ phosphorylation of STAT3	[35]
	mice C57BL/6		
	ovarian cancer cell line (SKOV3), monocytes (THP-1)	↓ phosphorylation of STAT3	[62]
	glioblastoma stem cell line (CD133+)	↓ phosphorylation of STAT3 ↓ expression of STAT3 ↓ nuclear translocation of STAT3	[53]
	prostate cancer cell line (DU145)	↓ phosphorylation of STAT3 ↓ STAT3 DNA-binding activity ↓ nuclear level of STAT3	[58]
Flavokawain B *Piper methysticum*	hepatocellular carcinoma cell line (HepG2)	↓ mRNA level of STAT3	[52]
Geranyl dihydrochalcone *Artocarpus altilis*	prostate cancer cell line (DU145)	↓ phosphorylation of STAT3	[63]
Isoliquiritigenin *Glycyrrhiza glabra*	breast cancer cell lines (Hs-578T and MDA-MB-231)	No changes in phosphorylation of STAT3 ↓ STAT3 DNA-binding activity ↑ PIAS3 level	[59]
	multiple myeloma cell line (U266)	↓ phosphorylation of STAT3	[41]
	Caki renal carcinoma cell line	↓ phosphorylation of STAT3	[56]
Licochalcone A *Glycyrrhiza glabra*	hematopoietic cell line (Ba/F3)	↓ phosphorylation of STAT3 ↓ nuclear translocation of STAT3	[64]
Licochalcone B *Glycyrrhiza glabra*	esophageal cancer cell lines (KYSE450 and KYSE510)	↓ phosphorylation of STAT3 ↓ STAT3 DNA-binding activity	[55]
Licochalcone C *Glycyrrhiza glabra*	oral squamous cell carcinoma lines (HN22 and HSC4)	↓ phosphorylation of STAT3	[36]
Licochalcone D *Glycyrrhiza glabra*	oral squamous cell carcinoma lines (HN22 and HSC4)	↓ phosphorylation of STAT3	[37]
Licochalcone H	skin cancer cell lines (A375 and A431)	↓ phosphorylation of STAT3	[38]
	oral squamous cell carcinoma cell lines (HN22 and HSC4)	↓ phosphorylation of STAT3	[65]
Phloretin *Manchurian apricot*	hepatocellular carcinoma cell lines (HepG2, SK-Hep1, Hep3B2.1-7, Huh7 and PLC-5)	↓ phosphorylation of STAT3 ↓ STAT3 activity	[39]
	mice xenografts (HepG2SR and Huh7SR)		
	pancreatic cancer cell lines (PaTu-8988T and PANC-1)	↓ phosphorylation of STAT3	[66]
Xanthohumol *Humulus lupulus*	breast cancer cell line (MCF-7) adriamycin (doxorubicin)-resistant breast cancer cell line (MCF-7/ADR)	↓ expression of STAT3	[50]
	choliangiocarcinoma cell lines (M139 and M214)	↓ expression of STAT3	[51]
	mice xenografts (KKU-M214)		
(E)-1–(1-hydroxy-4,5,8-trimethoxynaphthalen-2-yl)-3-(quinolin-6-yl) prop-2-en-1-one	gastric cancer cell line (MKN1)	↓ phosphorylation of STAT3	[43]
(E)-3-(7-(3,4-dimethoxyphenyl)-2- phenylpyrazolo[1,5-a]pyrimidin-5-yl)-1- (3,4,5-trimethoxyphenyl) prop-2-en-1-one	lung cancer cell line (A549)	↓ phosphorylation of STAT3	[45]
(E)-1-(2,4-dimethoxyphenyl)-3-(4-hydroxy-3,5-dimethoxyphenyl) prop-2-en-1-one	breast cancer cell line (MDA-MB-231)	↓ phosphorylation of STAT3	[46]
(E)-3-(4-bromo-3,5-dimethoxyphenyl)-1-(3-hydroxyphenyl) prop-2-en-1-one	melanoma cell lines (Sk-Mel-5 and Sk-Mel-28)	↓ phosphorylation of STAT3	[42]
N-(4-(3-(4-methoxyphenyl)acryloyl) phenyl)-2-((5-(3,4,5-trimethoxy- phenyl)-1,3,4-oxadiazol-2-yl)thio) acetamide	leukemia cell line (K-562)	↓ STAT3 activity	[60]
4,3′,4′,5′-tetramethoxychalcone	ovarian cancer cell lines (A2780 and SKOV3)	↓ phosphorylation of STAT3	[44]
ZE-2-(4-(4-chlorophenyl)-6-(4-nitrophenyl)pyrimidin-2-ylthio)- N-(4-(3-(3,4-dimethoxyphenyl) acryloyl)phenyl)acetamide	pancreatic cancer cell line (PANC-1)	↓ phosphorylation of STAT3	[47]
3-(4-methylthiophene)-1-(3-bromo-4,5-dimethoxyphenyl)prop-2-en-1-on and 3-(3-methoxy-4-methylthiophenyl)-1- (3-bromo-5-methoxy-4- methylthiophen)prop-2-en-1-on	colorectal carcinoma cell lines (DLD-1 and HCT116)	↓ phosphorylation of STAT3 ↓ nuclear levels of STAT3 ↓ binding of STAT3 to DNA	[48]

↑/↓ increase/decrease.

## 4. NF-κB and Chalcones

### 4.1. NF-κB Signaling Pathways as a Target

NF-κB (nuclear factor kappa B) is a transcription factor that plays a crucial role in regulating various cellular processes, including immune responses, inflammation, cell survival, and development. The canonical pathway of NF-κB activation involves the activation of the classical IKK (IκB kinase) complex and the subsequent release of NF-κB dimers from inhibitory IκB proteins. This process leads to the translocation of NF-κB into the nucleus, regulating the expression of the target genes [67]. The canonical NF-κB pathway can be activated by a wide range of stimuli, including pro-inflammatory cytokines (such as tumor necrosis factor alpha, TNF-α and interleukin 1 beta, IL-1β), microbial products (such as lipopolysaccharide, LPS), and cellular stressors (such as oxidative stress or DNA damage). These stimuli are recognized by various receptors, such as tumor necrosis factor receptor (TNFR), toll-like receptors (TLRs), or interleukin-1 receptors (IL-1Rs), which initiate the signaling cascade, leading to the activation of the IKK complex. The classical IKK complex consists of two catalytic subunits, IKKα and IKKβ, and a regulatory subunit, IKKγ (also known as NEMO). Activated IKK phosphorylates IκB proteins, primarily IκBα, targeting them for degradation by the proteasome. IκB proteins function as inhibitors by binding to NF-κB dimers and sequestering them in the cytoplasm. The phosphorylation of IκBα triggers its ubiquitination and subsequent degradation, releasing NF-κB dimers [68]. NF-κB dimers (commonly the p50/p65 heterodimer) translocate into the nucleus and bind to specific DNA sequences called κB sites, promoting the transcription of target genes involved in immune responses, inflammation, cell proliferation, cell survival, and other biological processes. NF-κB recruits coactivators, corepressors, and other transcriptional regulators to modulate gene expression.

Besides the canonical pathway, there are non-canonical pathways of NF-κB activation that involve different signaling mechanisms and can contribute to distinct cellular responses. The non-canonical NF-κB pathway is primarily associated with processing the p100 precursor protein, also known as NF-κB2, and the subsequent generation of the active p52 subunit. The non-canonical pathway is typically triggered by a subset of tumor necrosis factor (TNF) family members, such as the B-cell activating factor (BAFF), CD40 ligand (CD40L), or lymphotoxin-β. The non-canonical NF-κB pathway involves the activation of a specific kinase complex called the non-canonical IκB kinase (IKK) complex, composed of NF-κB-inducing kinase (NIK), an inhibitor of NF-κB kinase α (IKKα), and NF-κB essential modulator (IKKγ), also known as NEMO [69]. Under basal conditions, NIK is constitutively synthesized but rapidly degraded through the action of TNF receptor-associated factor 3 (TRAF3), which promotes its ubiquitination and subsequent proteasomal degradation. Upon the activation of the non-canonical NF-κB pathway, specific TNF receptor family members activate downstream signaling cascades that stabilize and accumulate NIK. The accumulated NIK then phosphorylates and activates IKKα, which subsequently phosphorylates p100. This phosphorylation event triggers the proteasomal processing of p100 into p52. The liberated p52 subunit then forms a complex with RelB (a distinct member of the NF-κB family) and translocates into the nucleus to regulate target gene expression such as Cyclooxygenase-2 (COX-2), interleukin (Il-6), and B-cell lymphoma 3-encoded protein (BCl3) [70]. 

It is crucial to acknowledge that the dysregulation of the non-canonical pathway has also been linked to the development and progression of certain cancers. 

### 4.2. Chalcones as NF-κB Inhibitors

Recent studies indicate that chalcones are also being investigated regarding their action on NF-κB activation. The anti-inflammatory properties of chalcones depend on their structure and the type of activation of this pathway, consequently binding to target proteins. Based on the literature data, it has been demonstrated that functional groups or stereochemistry in chalcones may facilitate interactions with proteins involved in inflammation, influencing the activity of the NF-kB pathway. Moreover, chalcones can bind to specific proteins that activate inflammatory pathways, inhibiting their function and suppressing the inflammatory response. These biological activities are mainly attributed to the presence of an α,β-unsaturated carbonyl system in chalcones, perceived as a potential acceptor of the Michael reaction (leading to the forming of a new carbon–carbon bond). This moiety can readily form covalent bonds with nucleophiles, such as thiol cysteine residues in peptides or cellular proteins, to obtain a Michael adduct, which may play an essential role in the molecule’s biological activity [13]. Among the potential molecular targets of anticancer therapies, the NF-κB pathway is characteristic due to its complexity and interaction with multifunctional proteins. In this regard, chalcones also demonstrate the ability to control inflammation by inhibiting NF-ĸB binding to DNA [71].

**Figure 4:**
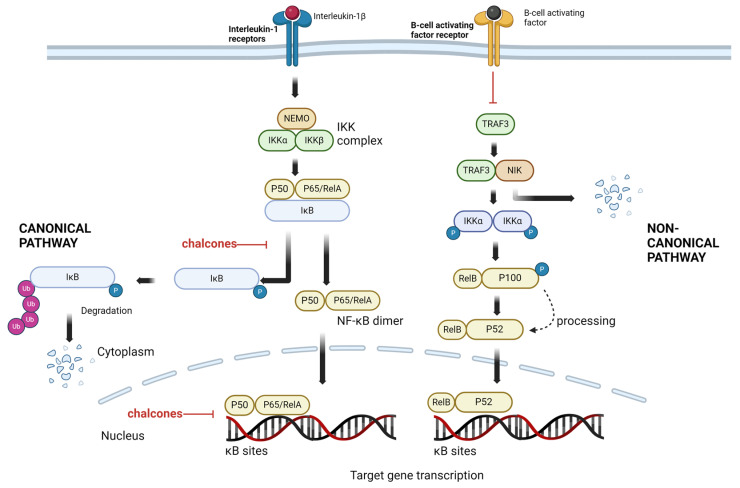
The proposed mechanism of chalcones on the inhibition of NF-κB activation. The left part of the figure represents the canonical pathway of NF-κB activation, the right part, the non-canonical pathway of NF-κB activation. Chalcones can block the NF-κB pathway by inhibiting the degradation of IκB or interfering with the DNA-binding activity of NF-κB. This figure was created using information from the references given in Section 4 (NF-κB and chalcones). κB site, specific DNA sequences binding NF-κB; IκB, inhibitor of kappa; IKKα, inhibitor of NF-κB kinase α; IKKβ, inhibitor of NF-κB kinase β; NEMO, NF-κB essential modulator; NF-κB, nuclear factor kappa B; TRAF3, TNF receptor-associated factor 3; NIK, NF-κB-inducing kinase; P, phosphorylation; P50, subunit of NF-κB; P65/RelA, subunits of NF-κB, P52, subunit of NF-κB; P100, subunit of NF-κB; RelB, subunit of NF-κB transcription factors; Ub, ubiquitination. Created with BioRender.com.

Many studies, leveraging information from diverse databases (PubMed, Scopus, Web of Science, and Google Scholar), have demonstrated that chalcones can suppress NF-κB activation through multiple mechanisms presented in Figure 4 and Table 2.

Firstly, chalcones inhibit the degradation of IκB (inhibitor of κB), an inhibitor protein that sequesters NF-κB in the cytoplasm. By preventing the phosphorylation and subsequent proteasomal degradation of IκB, chalcones effectively block NF-κB translocation to the nucleus, thereby inhibiting its transcriptional activity. An example of such a chalcone is butein, which inhibits the IKK activity, subsequently suppressing the phosphorylation of IκBα and degradation in a myelogenous leukemia cell line (KBM-5) and a multiple myeloma cell line (U266) [72].

Under the influence of cardamonin, the level of IKKα/β, IKKβ, and the phosphorylation of IκBα decreased in the nasopharyngeal carcinoma cell line (CNE-2) [73]. A decreased level of phosphorylated IκBα was also observed in treatment with isoliquiritigenin [74] licochalcone A [75], licochalcone B [76], and phloretin [77] treatment. It is also reported that synthetic chalcones can act in the same way. The studies indicated that treatment with 2-hydroxy-3′,5,5′-trimethoxychalcone leads to a decrease in IKKα/β and IĸB phosphorylation in the breast cancer cell line (MDA-MB-231) [78], and 2′,4′,6′-tris(methoxymethoxy) chalcone restrains the degradation of IĸBα in pancreatic acinar cells from the C57BL/6 mice [79].

Secondly, chalcones can directly interfere with the DNA-binding activity of NF-κB. In pancreatic cancer cells (PANC-1), xanthohumol decreased the binding to DNA in both subunits of NF-κB p50 and NF-κB p65 [80]. For licochalcone A, the inhibition of binding subunit NF-κB p65 only to DNA was demonstrated in the hepatocellular carcinoma cell line (SK-Hep-1) [75]. In turn, for the in vivo model, reduced binding NF-κB to DNA was noted for phloretin [77].

When the NF-κB dimers (commonly the p50/p65 heterodimer) translocate into the nucleus and bind to specific DNA sequences called κB sites, they promote the transcription of target genes involved in immune responses (e.g., IL-6), inflammation (e.g., COX-2), cell proliferation (e.g., c-Myc), cell survival (e.g., cyclin D1), angiogenesis (e.g., VEGF), and other biological processes. In the case of butein, a dose-dependent reduction in COX-2 and Matrix metalloproteinase-9 (MMP-9) expression in oral squamous cell carcinoma lines (SAS and KB) was observed [81]. The other study conducted by Pandey et al. has indicated that butein decreased c-Myc, cyclin D1, COX-2, MMP-9, and VEGF gene products in a myelogenous leukemia cell line (KBM-5) and multiple myeloma cell line (U266) [72]. Whereas, for cardamomin in the in vitro and in vivo models, the inhibition of genes such as Mcl-1, cyclin E, and Bcl-2 proteins and the induction of Bax proteins were observed [73,82,83]. In turn, a decrease in COX-2 expression was observed in both the in vitro and in vivo models for phloretin [77,84]. Meanwhile, for xanthohumol, a dose-dependent decrease in COX-2 levels was demonstrated in the pancreatic cancer cell line (PANC-1] [80]. Furthermore, xanthohumol has been shown to decrease the expression of VEGF and IL-8 both in vitro and in vivo [85].

**Table 2:** Summary of natural and synthetic chalcones acting on NF-κB pathways, the model used, and results obtained.

Name of Chalcones	In Vitro/In Vivo Models	Effect on NF-κB	References
Butein *Semecarpus anacardium*	myelogenous leukemia cell line (KBM-5), multiple myeloma cell line (U266)	↓ IKK activity ↓ phosphorylation and degradation of IκBα ↓ NF-κB p65 activity	[72]
	oral squamous cell carcinoma line (KB) tongue squamous cell carcinoma (SAS)	↓ NF-κB activity	[81]
	prostate cancer cell line (LNCaP)	↓ level of NF-κB, IKKα ↓ phosphorylation and degradation of IκBα	[86]
Cardamonin *Alpinia rafflesiana*	ovarian cancer cell line (SKOV3)	↓ phosphorylation of NF-κB ↓ level of NF-κB, IKKα/β, IKKβ	[87]
	mice BALB/c		
	hepatoblastoma cell line (HepG2)	↓ phosphorylation of NF-κB p65 ↓ level of IKKβ	[82]
	ICR mice		
	nasopharyngeal carcinoma cell line (CNE-2)	↓ nuclear level of NF-κB p65 ↓ phosphorylation of NF-κB p65 ↓ level of IKKα/β ↓ phosphorylation of IκBα	[73]
	colon cancer cell line (5-FU-resistant HCT116)	↓ level of NF-κB p65	[83]
Isoliquiritigenin *Glycyrrhiza glabra*	hepatoblastoma cell line (HepG2)	↓ nuclear level of NF-κB p65 ↑ level of IĸBα ↓ phosphorylation of IκBα ↓ NF-κB nuclear activity	[74]
Isoliquiritigenin 2′-methyl ether *Caesalpinia sappan*	oral squamous cell carcinoma cell lines (HN4 and HN12)	↑ phosphorylation of IκBα ↑ degradation of IκBα ↑ NF-κB p65 nuclear activity	[88]
Licochalconce A *Glycyrrhiza glabra*	hepatocellular carcinoma cell line (SK-Hep-1)	↓ nuclear level of NF-κB p65 ↓phosphorylation of IκBα	[75]
Licochalconce B *Glycyrrhiza glabra*	bladder carcinoma cell line (T24)	↓ phosphorylation of NF-κB p65 ↓ nuclear level of NF-κB p65 ↓ phosphorylation of IκBα	[76]
Phloretin *Manchurian apricot*	ICR mice with skin carcinogenesis	↓ DNA binding of NF-κB	[77]
	lung epithelial cell line (A549)	↓ NF-κB p65 translocation into the nucleus ↑nuclear level of NF-κB p65 ↓ phosphorylation and degradation of IκBα	[84]
Xanthohumol *Humulus lupulus*	pancreatic cancer cell line (PANC-1)	↓ mRNA level of NF-κB p65 and NF-κB p50 ↓ nuclear level of NF-κB p65 ↓ NF-κB p65 activity	[80]
	pancreatic cancer cell lines (BxPC-3, MIA PaCa-2 and AsPC-1)	↓ NF-κB p65 activity	[85]
	mice BALB/c		
	hepatoblastoma cell line (HepG2)	↓ level of NF-κB	[89]
α-2-bromo-N-(1-methyl-3-(3-oxo-3-(pyridin-4-yl)prop-1-en-1-yl)-1H-indol-5-yl)acrylamide	human melanoma cell line (SK-MEL-1)	↓ phosphorylation of NF-κB p65	[90]
2-hydroxy-3′,5,5′-trimenthoxychalcone	breast cancer cell line (MDA-MB-231)	↓ phosphorylation of NF-κB p65 ↓ phosphorylation of IKKα/β and IĸB ↓ nuclear level of NF-κB p65 ↓ NF-κB activity	[78]
2′-hydroxy-4-methylsulfonylchalcone and 4′-chloro-2′-hydroxy-4- methylsulfonylchalcone	prostate cancer cell line (PC-3)	↓ NF-κB nuclear activity	[91]
2′,4′,6′-tris(methoxymethoxy) chalcone	pancreatic acinar cells from the C57BL/6 mice	↓ degradation of IĸBα ↓ NF-κB activity	[79]
(2E,2′E)-1,1′-(5,5′-(piperazine-1,4-diylbis(methyl ene))bis(4-hydroxy-3-methoxy-5,1-phenylene))bis(3-ph enylprop-2-en-1-one)	nasopharyngeal carcinoma cell line (NPC-TW 039)	↓ phosphorylation of NF-κB ↓ nuclear level of NF-κB p65	[92]
3-(4-methylthiophene)-1-(3-bromo-4,5-dimethoxyphenyl)prop-2-en-1-on and 3-(3-methoxy-4-methylthiophenyl)-1-(3-bromo-5-methoxy-4-methylthiophene)prop-2-en-1-on	colorectal carcinoma cell lines (DLD-1 and HCT116)	↓ level of the nuclear level of NF-κB p50 and sNF-κB p65 ↓ transcript level of NF-κB p50 and NF-κB p65	[48]

↑/↓ increase/decrease.

In experimental models, inhibitors have been used to explain the activation mechanism of NF-ĸB. A study by Li et al. indicated a significant reduction in nuclear NF-ĸB p65 protein in CNE-2 cells after treatment with cardamomin for 12 h. Consistently, the phosphorylation of NF-ĸB p65 was inhibited in a time- and concentration-dependent manner. In addition, treatment with cardamonin showed a significant decline in the phosphorylation of IκBα and the expression of IKKα and IKKβ in as early as six hours. These results demonstrated that cardamonin inhibited the activity of the NF-κB pathway. As mentioned earlier, oxidative stress is a factor activating the NF-κB pathway. Li et al.’s research has shown that the inhibition of the NF-κB pathway was accompanied by increased reactive oxygen species (ROS) accumulation. Additionally, intracellular ROS levels were measured in the presence and absence of the NF-κB activator, TNF-α.

Similarly, cell viability was examined in the presence or absence of TNF-α during cardamomin treatment. The results of these studies showed that the reduced cell viability induced by cardamomin was partially attenuated after exposure to TNF-α. Studies also indicated that in the absence of TNF-α, cardamomin treatment showed a significant increase in ROS accumulation, whereas the rise in ROS accumulation induced by cardamomin was partially blocked in the presence of TNF-α. To confirm the NF-κB pathway’s regulatory impact on the ROS increase caused by cardamonin, nasopharyngeal carcinoma cells (CNE-2) were additionally exposed to NF-κB inhibitors like BAY 11-7082 and MG132. The intracellular ROS levels were evaluated in these samples using the Dichloro-dihydro-fluorescein diacetate (DCFH-DA) assay and the Dihydroethidium (DHE) assay. Taken together, cardamonin induced ROS accumulation via the inhibition of the NF-κB pathway [73].

The literature review did not reveal non-canonical activation of the NF-κB pathway under the influence of the presented chalcones in this paper. A detailed understanding of the mechanism requires extensive research to precisely identify chalcones as modulators of canonical and non-canonical NF-κB activation pathways. Such research would allow for the precise indication of chalcones for therapeutic purposes.

## 5. Targeting the NF-κB and STATs/STAT3 Pathways by Chalcones and Their Other Combination

The analysis of the literature data showed that, so far, only a few chalcones have been shown to act on both the STAT and NF-κB pathways in the same experimental model. This group includes xanthohumol, which may be a target STAT3 via the Akt-NF-κB signaling pathway, which leads to the inhibition of the proliferation of cholangiocarcinoma cells KKU-M139 and KKU-M214. Moreover, a reduction in the activity of STAT3 with 50 µM xanthohumol prominently decreased cell growth and enhanced apoptosis [51]. Studies on butein’s effects on the myeloma cell line (U266) indicated that it inhibits the phosphorylation of STAT3 [61] and reduces NF-κB activity in a dose-dependent manner [72].

In another study, butein inhibits pSTAT3(Y705) phosphorylation, the nuclear localization of NF-κB, and the physical interaction of NF-κB and pSTAT3 in Malignant Pleural Mesothelioma (MPM) cells. This correlates with the downregulation of several genes involved in the cancer progression (such as MMP-9) of proangiogenic cytokines (VEGF) and of IL-6 and IL-8, key growth factors for MPM [93]. Research by Jia et al. showed that cardamonin decreased the activation of STAT3 and NF-κB in breast cancer cell lines (SUM190, MCF-7). Additionally, they demonstrated that cardamonin abrogated the chemotherapeutic doxorubin-induced activation of NF-κB and STAT3 [94].

One approach that has shown promise is the development of combination therapies using chalcones with other polyphenols. These combinations have been investigated for their ability to simultaneously inhibit both STAT and NF-κB pathways, leading to synergistic effects and enhanced therapeutic outcomes. An example is the study by Nourbakhsh et al., which used a combination of a novel chalcone derivative 1-(4-(methylsulfonyl)phenyl)-3-(phenylthio)-3-(p-tolyl)propane-1-one with curcumin, a well-known herbal medicine. The researchers found that this combination effectively inhibited the DNA-binding activity of NF-κB in the breast cancer cell line (MDA-MB-231) and ovarian cancer cell line (SKOV3) and subsequently suppressed the expression of downstream genes, including COX-2, MMP-9, and inducible nitric oxide synthase (iNOS). 1-(4-(methylsulfonyl)phenyl)-3-(phenylthio)-3-(p-tolyl)propane-1-one and its coadministration with curcumin effectively reduced the activity of the NF-κB signaling pathway, leading to a reduced inflammatory response in the environment of cancer cells, and might be considered an effective remedy for the suppression of inflammatory processes in MDA-MB-231 and SKOV3 cell lines [95]. Another example is the study by Cykowiak et al., which showed that the combination of xanthohumol and phenethyl isothiocyanate decreased STAT3 levels and the activation of NF-κB and subsequently reduced COX-2 in pancreatic cancer cells (PSN-1), which indicated their anti-inflammatory and pro-apoptotic activities [96].

Other studies have indicated a more substantial therapeutic potential of the combination of chalcones with drugs, investigating the effects of the combination of daunorubicin (DNR) and flavokawain B (FKB) on human leukemic cells. The combination treatment of DNR and FKB may improve the anticancer effects of DNR in patients with DNR-resistant leukemia. FKB induces apoptosis through changes in NF-ĸB activation and the phosphorylation subunit p-p65 [97].

Literature data from the database (PubMed) indicate the influence of chalcone on radiotherapy and chemotherapy (Table 3). An in vitro model showed increased sensitivity to chemotherapy as a result of the synergistic effect of chemotherapy with butein, phloretin, isoliquiritigenin, xanthohumol, and isoxanthohumol [40,98,99,100,101,102]. However, an increase in the sensitivity of stomach, lung, and breast cancer cells to radiotherapy was observed after the use of butein, phloretin, and xanthohumol, respectively [50,103,104]. Moreover, phloretin also enhanced the effect of radiotherapy in a C57BL/6J mouse model [104]. The results observed regarding the influence of chalcones on chemotherapy/radiotherapy primarily stemmed from the increased induction of apoptosis and/or the restriction of proliferation in the tested cancer cells.

These studies highlight the potential of combining chalcones as therapeutic agents against cancer and inflammatory disorders by simultaneously targeting the STAT and NF-κB pathways. However, it is important to note that further research is needed to optimize these compounds’ efficacy, safety, and pharmacokinetic properties before they can be translated into clinical applications.

**Table 3:** Summary of the synergistic effect of chalcones with chemo- and radiotherapy.

Combination	In Vitro/In Vivo Models	Synergistic Effect	References
Butein + radiotherapy	gastric cancer cell line (MKN-45)	↑ radiosensitivity	[103]
Butein + doxorubicin	liver cancer cell line (HepG2)	↑ chemosensitivity	[40]
Butein + paclitaxel			
Isoliquiritigenin + gemcitabine	pancreatic cancer cell lines (PANC1, MIA PaCa-2)	↑ chemosensitivity	[98]
Isoliquiritigenin +5-fluorouracil	gastric cancer cell line (MKN45) gastric cancer mice xenografts	↑ chemosensitivity	[99]
Licochalcone A + paclitaxel	squamous cell carcinoma cell line (SCC-15)	↑ chemosensitivity	[105]
Phloretin + tamoxifen	breast cancer cell lines (MCF7, MDA-MB-231)	↑ chemosensitivity	[100]
Phloretin + doxorubicin			
Phloretin + radiotherapy	Lewis lung cancer cell line	↑ radiosensitivity	[104]
	C57BL/6J mice		
Xanthohumol + radiotherapy	breast cancer cell line (MCF-7) adriamycin (doxorubicin)-resistant breast cancer cell line (MCF-7/ADR)	↑ radiosensitivity	[50]
Xanthohumol + doxorubicin		↑ chemosensitivity	[106]
Xanthohumol + 7-ethyl-10-hydroxycamptothecin	colorectal cancer cell line (SW480)	↑ chemosensitivity	[101]
Isoxanthohumol + paclitaxel	melanoma cell lines (B16 and A375)	↑ chemosensitivity	[102]
	C57BL/6 mice		

↑/↓ increase/decrease.

## 6. Targeting the Crosstalk between NF-κB and STAT3/STATs Pathways by Chalcones

Considering the previously discussed inhibitory effect on the NF-κB and STAT3/STATs pathways, we should also focus on the potential interactions. Emerging evidence suggests extensive crosstalk between the NF-κB and STAT pathways. The activation of NF-κB signaling can induce the expression of cytokines and growth factors that subsequently activate the JAK/STAT cascade. Conversely, activated STATs can enhance NF-κB activity by promoting the production of pro-inflammatory mediators. This reciprocal activation forms a positive feedback loop, amplifying inflammatory responses and contributing to disease progression [107]. However, no literature data indicate that chalcones accelerate the inflammatory process.

Many studies indicate the inhibitory potential of chalcones regarding the anti-inflammatory STAT and NF-κB pathways. Chalcones can inhibit NF-κB activation by suppressing IKK activity, preventing IκB degradation, or directly inhibiting the DNA-binding activity of NF-κB. Additionally, chalcones have been shown to inhibit JAK/STAT signaling by interfering with JAK activation or STAT phosphorylation. These dual inhibitory effects of chalcones on NF-κB and STAT pathways provide a unique strategy to attenuate inflammation and regulate immune responses [33].

Recently published studies examined the effect of eight newly synthesized thio-chalcones on the NF-κB and STAT3 signaling pathways in DLD-1 and HCT116 colorectal cancer cells. The tested thio-derivatives of chalcones such as 3-(4-methylthiophene)-1-(3-bromo-4,5-dimethoxyphenyl)prop-2-en-1-one and 3-(3-methoxy-4-methylthiophenyl)-1-(3-bromo-5-methoxy-4-methylthiophene)prop-2-en-1-one showed a cytotoxic effect because they inhibited the viability of colon cancer cells (DLD-1 and HCT116). Furthermore, the expression analyses showed reduced activity due to the NF-κB and STAT3 factors by attenuating the translocation from the cytosol to the nucleus and decreasing the protein level of controlled genes by studied chalcones [48].

Other interactions and forms of crosstalk between NF-κB and STAT3 include physical interaction between the two, cooperation of these factors at gene promoters/enhancers, the NF-κB-dependent expression of inhibitors of STAT3 activation, and the participation of STAT3 in inflammatory cells in the negative regulation of NF-κB. Despite these versatile and occasionally antagonistic interactions, NF-κB and STAT3 cooperate to promote colon, gastric, and liver cancer development and progression. In addition to explaining the molecular pathogenesis of cancer, these interactions also offer opportunities to design new therapeutic interventions [108].

Additionally, the ability of chalcones to simultaneously modulate these key signaling cascades offers new therapeutic opportunities for various inflammatory and immune-mediated diseases and cancers. However, further research is needed to elucidate the exact mechanisms of action of chalcones and their potential clinical applications.

## 7. Clinical Trials with Chalcones

Although chalcones are of great interest in the field of medicinal chemistry due to their diverse biological effects, only a detailed analysis of literature data (PubMed, ClinicalTrials.gov) allows us to draw attention to only a few examples of research conducted on chalcones in the context of clinical trials. Chalcones, such as metochalcone and sofalcone, have shown promising potential as therapeutic agents in clinical trials. Metochalcone has been approved as a choleretic drug, while sofalcone has demonstrated anti-ulcer properties by increasing mucosal prostaglandin levels, providing gastroprotective effects against *Helicobacter pylori* infection. These findings have paved the way for exploring chalcones as novel anticancer agents [109,110]. Recently, Jeong et al. and Oh et al. revealed that synthetic chalcones can inhibit heat-shock protein 90 (Hsp90), a protein involved in the survival and multiplication of cancer cells. A novel chalcone-based molecule, (E)-3-(2-bromo-3,4,5-trimethoxyphenyl)-1-(2,4-dihydroxyphenyl)prop-2-en-1-one (BDP), inhibits MDA-MB-231 triple-negative breast cancer cell growth by suppressing Hsp90 function [111,112]. Other studies using male, adult albino CD1 mice have indicated that the reduced level of Hsp90 protein results from suppressing STAT transcriptional activity [113,114].

Thus, this discovery offers new perspectives for cancer treatment by targeting proteins that contribute to tumorigenesis. Phase II clinical trials investigating chalcones with hydroxyl groups at positions 1 and 3 have demonstrated the inhibition of Hsp90 interactions with patient proteins through binding to the ATP site in Hsp90. These positive results suggest the potential of chalcones and their derivatives as anticancer agents, and it is conceivable that future phase III clinical trials will further support their efficacy [115]. An early phase 1 study examined the effects of licochalcone A and paclitaxel on the human oral squamous cell carcinoma cell line (SSC-15). The combination treatment of licochalcone A and paclitaxel showed the maximum antiproliferative effect on SSC-15 through downregulating the IPO-38 proliferation marker, which consequently resulted in the increased sensitivity of cells to the chemotherapeutic agent paclitaxel [105].

Chalcones hold promise as novel therapeutic agents targeting certain factors involved in the NF-κB and STAT3 signaling pathways in treating inflammatory diseases and cancer. Further well-designed clinical trials are warranted to establish the optimal dosage, safety profile, and long-term effectiveness of chalcones in inhibiting NF-κB and STAT3 activation.

## 8. Nanoformulations as a Future of Chalcones

Nanoformulations represent a promising avenue for enhancing the therapeutic potential of chalcones, a class of natural compounds known for their diverse pharmacological activities. Chalcones can be encapsulated in nano-sized carriers by utilizing nanotechnology, improving their solubility, stability, and bioavailability. This approach addresses challenges associated with the limited water solubility of chalcones, enhancing their delivery and efficacy in various biomedical applications. Examples of nanoformulations of chalcones include lipid-based nanoparticles, polymeric nanoparticles, and micelles. These carriers provide a protective environment for chalcones, preventing degradation and improving their absorption in the body. Additionally, nanoformulations enable targeted drug delivery, enhancing the specificity of chalcones toward diseased cells while minimizing the side effects on healthy tissues. One of the directions of research conducted using chalcones is to improve their chemical structure to increase their effectiveness and safety of use and support targeted therapies. These studies include developing nanoformulations delivering natural chalcones and/or their derivatives to specific cells to increase their capabilities in clinical applications. An example of such modifications is the developed poly-lactic-co-glycolic acid (PLGA) nanoparticles encapsulating xanthohumol and tested for antiproliferative, anticancer, and migratory effects on malignant skin melanoma cells (B16F10) and mouse macrophages (RAW 264.7). The obtained results confirm the anticancer effect of PLGA nanoparticles loaded with xanthohumol and represent the first signs of progress towards the use of a nanoformulation delivering xanthohumol to reduce the side effects of currently used chemotherapeutics [116].

Moreover, to overcome the limitations of xanthohumol bioavailability, a loading system formulation of xanthohumol-loaded solid lipid nanoparticles with a sustained xanthohumol release profile after oral administration was successfully demonstrated without losing its anticancer properties against prostate adenocarcinoma-derived cells (PC-3) [117]. In turn, research by Sun, 2017, described a drug delivery system (hollow gold nanoparticles) for the slightly water-soluble natural anticancer licochalcone A isolated from *Glycyrrhiza inflata* [118]. Anticancer properties have also been confirmed for nanoliposomes containing isoliquiritigenin. Research by Wang et al. confirmed the potential for their use as an adjuvant treatment for colorectal cancer [119].

The cited studies indicate that drug delivery systems based on nanoparticles are a new way to increase chalcones’ effectiveness in treating various types of cancer.

## 9. Conclusions

In conclusion, targeting the STAT3 and NF-κB signaling pathways in cancer prevention and treatment holds great promise, and chalcones have emerged as potential therapeutic agents in this regard. The STAT and NF-κB pathways play crucial roles in tumor initiation, progression, and metastasis, making them attractive targets for intervention. Chalcones, a class of natural compounds found in various plant sources, have shown significant potential in modulating these signaling pathways, thereby exerting anticancer effects. Studies have demonstrated that chalcones can inhibit the activation of STAT3 and NF-κB, suppressing genes involved in cell survival, proliferation, angiogenesis, and inflammation. By targeting these pathways, chalcones exhibit multiple mechanisms of action, including the induction of apoptosis, cell cycle arrest, the inhibition of metastasis, and the modulation of immune responses. These properties make chalcones attractive candidates for cancer prevention and treatment. Moreover, chalcones have demonstrated promising synergistic effects with conventional cancer therapies, such as chemotherapy and radiotherapy. They can enhance the cytotoxic effects of these treatments while reducing their side effects, thereby improving therapeutic outcomes.

Moreover, chalcones have demonstrated promising synergistic effects with conventional cancer therapies, such as chemotherapy and radiotherapy. They can enhance the cytotoxic effects of these treatments, increase radiosensitivity and chemosensitivity, and at the same time reduce their side effects, thus improving therapeutic outcomes.

Moreover, chalcones have shown selectivity towards cancer cells, sparing normal cells and enhancing their therapeutic potential. Although chalcones hold significant promise, further research is needed to fully understand their molecular mechanisms of action and optimize their efficacy and safety profiles. Clinical trials are necessary to evaluate their effectiveness in humans and determine the appropriate dosage and administration regimens.

In conclusion, targeting the STAT3 and NF-κB signaling pathways using chalcones represents a promising cancer prevention and treatment approach. Chalcones offer a natural and multifaceted strategy to disrupt critical signaling pathways in cancer pathogenesis. With continued research and development, chalcones may hold great potential as a valuable addition to the armamentarium of anticancer therapies, providing new opportunities for improved patient outcomes in the fight against cancer.
